# Case Report: Rapid Treatment of Uridine-Responsive Epileptic Encephalopathy Caused by CAD Deficiency

**DOI:** 10.3389/fphar.2020.608737

**Published:** 2020-12-07

**Authors:** Ling Zhou, Jie Deng, Sarah L. Stenton, Ji Zhou, Hua Li, Chunhong Chen, Holger Prokisch, Fang Fang

**Affiliations:** ^1^ Department of Neurology, Beijing Children's Hospital, Capital Medical University, National Center for Children's Health, Beijing, China; ^2^ Institute of Human Genetics, Technische Universität München, München, Germany; ^3^ Institute of Neurogenomics, Helmholtz Zentrum München, München, Germany

**Keywords:** CAD deficiency, epilepsy, anaemia with anisopoikilocytosis, uridine, developmental delay

## Abstract

We present two unrelated Chinese patients with CAD deficiency manifesting with a triad of infantile-onset psychomotor developmental delay with regression, drug-refractory epilepsy, and anaemia with anisopoikilocytosis. Timely translation into uridine supplementation, within 2-months of disease onset, allowed us to stop conventional anti-epileptic drugs and led to dramatic improvement in the clinical symptoms, with prompt cessation of seizures, resolution of anaemia, developmental progress, and prevention of development of severe and non-reversible manifestations. The remarkable recovery and prevention of advanced disease with prompt treatment, highlights the need to act immediately upon genetic diagnosis of a treatable disease. This further reinforces CAD deficiency as a treatable neurometabolic disorder and emphasises the need for a biomarker or genetic new born screening for early identification.

## Introduction

A trifunctional protein (carbamoyl phosphate synthetase, asparatate transcarbamylase and dihydroorotase) encoded by CAD, enzymatically accounts for the first three steps of the six-step *de novo* pyrimidine synthesis (Chen et al., 1989). Dysfunctional CAD results in pyrimidine deficiency, and corresponds clinically to EIEE-50 (early infantile epileptic encephalopathy 50, OMIM# 114010). This deficiency can be compensated by an alternative mechanism, whereby pyrimidine is recycled from uridine, giving rise to the treatment option of uridine supplementation with antiepileptic effect attributed to the salvage pathway (Nyhan, 2005). Following the discovery of the first patient with biallelic CAD mutations in 2015, and with a more recent retrospective series published of 20 subjects in Rymen et al. (2020) Genetic Medicine 2020, there remains a lack of detailed depictions of the clinical picture of CAD deficiency prior to or post uridine treatment (Ng et al., 2015; Koch et al., 2017; Del Caño-Ochoa et al., 2020; Rymen et al., 2020; Zhou et al., 2020).

Here, we report two further unrelated Chinese patients with CAD deficiency presenting with a triad of infantile-onset psychomotor developmental delay with regression, drug-refractory epilepsy, and anaemia with anisopoikilocytosis. In addition to the phenotypes reported to date, we report optic nerve involvement with clinical signs raising suspicion of visual impairment. Diagnostic whole exome sequencing (WES) was initiated within 1-month of disease onset, and within 2-weeks of identifying the genetic diagnosis. This prompted immediate supplementation of uridine, achieving compelling improvement in the above clinical symptoms and prevention of the development of severe and non-reversible manifestations. This therapeutic success further supports the designation of CAD deficiency as a treatable epileptic encephalopathy and neurometabolic disorder, amongst a limited number of other notable exceptions (Dulac et al., 2014; van Karnebeek et al., 2014; Tarailo-Graovac et al., 2016).

## Case Report

**FIGURE 1:**
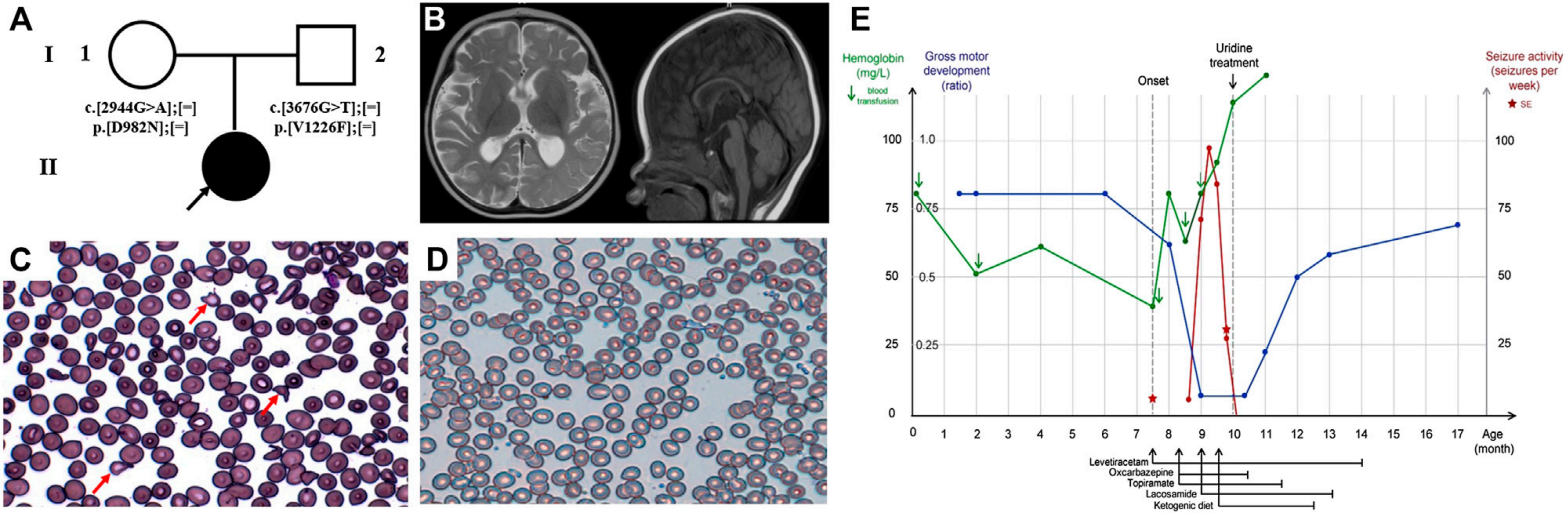
Clinical features and uridine response of patient 1 with CAD deficiency. **(A)** Family pedigree of patient 1. **(B)** Brain MRI at 10-months. Peripheral blood smear **(C)** before treatment and **(D)** after treatment. **(E)** Clinical overview during the course of disease. Gross motor development represented as the observed development age according to the Nelson Gross Motor Development Milestones divided by the patient’s biological age (Feigelman, 2016).

Patient 1 is a girl currently 17-months of age. She is the first child of a healthy nonconsanguineous couple and was exclusively formula milk fed (Figure 1A). She received her first blood transfusion due to unexplained anaemia within one week of birth. Her neonatal period was otherwise uncomplicated. At the age of 7.5-months, she presented with recurrent afebrile seizures. The seizures were mostly of focal onset, with several episodes of status epilepticus, manifesting as eyes slanted to one side with ipsilateral or bilateral upper limb slightly shaking, lasting from several minutes to 1 h. She demonstrated global development delay prior to onset of the seizure attacks, and following seizure onset, she gradually lost head control, becoming hypotonic. Her brain MRI at 10-months showed mildly delayed myelination of the white matter, accompanied by mild atrophy-like change (Figure 1B). The interictal VEEG performed at 9-months recorded sharp and sharp-slow wave discharges on the left mid-to-posterior temporal lobe, while the ictal EEG documented 11 attack events (eyes turned right with slightly shaking of four limbs) originating from the left posteromedial temporal lobe (Supplementary Figure S1A). In addition, she presented with moderate to severe anaemia (haemoglobin 4‒6.7, reference 11–16 g/dl; mean corpuscular volume 75.5–84.1, reference range 80–100 fl), necessitating five blood transfusions. The peripheral blood smear suggested abnormal erythrocytes of varying size and abnormal morphology (Figure 1C). Visual evoked potential (VEP) at 11-months suggested failure to evoke P100 wave, indicative of an optic nerve conduction block, with a normal examination of the fundus. Several anti-epileptic drugs (AEDs), including levetiracetam (LEV 50 mg/kg day), oxcarbazepine (OXC 50 mg/kg day), topiramate (TPM 4.5 mg/kg day), lacosamide (LCM 4.5 mg/kg day) and nitrazepam (NZP 0.25 mg/kg day), in addition to ketogenic diet therapy at a ratio of 2:1 were trialled for 1.5-months. These interventions, however, achieved poor efficacy. She achieved a seizure-free period for 1 month when taking LEV (20 mg/kg day), however her seizures subsequently recurred up to approximately 10 times per day. Two months after the first seizure, and just 2-weeks after identification of the CAD variants, she was administered oral uridine (100 mg/kg daily). Seizure cession was observed by day 2 of uridine administration. She stopped taking AEDs (OXC, TPM, KD, LCM, and LEV in order) approximately 2-months after commencing uridine and has remained seizure-free for 8 months follow-up to-date. Since treatment, a sequential interictal VEEG performed at 11-months of age recorded no epileptiform discharge, and the background activities were normal for the age (Supplementary Figure S1B). Re-examination of routine blood tests 1.5-months after commencing uridine supplementation demonstrated normalised haemoglobin as well as erythrocytes morphology, and the peripheral blood smear was almost normalised (Figure 1D). Re-examined VEP at 1-year of age demonstrated slightly decreased amplitude of P100 and prolonged latency. Moreover, she has made significant progress in development, and is now able to walk with assistance and speak several words, such as “mama,” “papa,” and “give me.” Her clinical course is depicted in Figure 1E.

**FIGURE 2:**
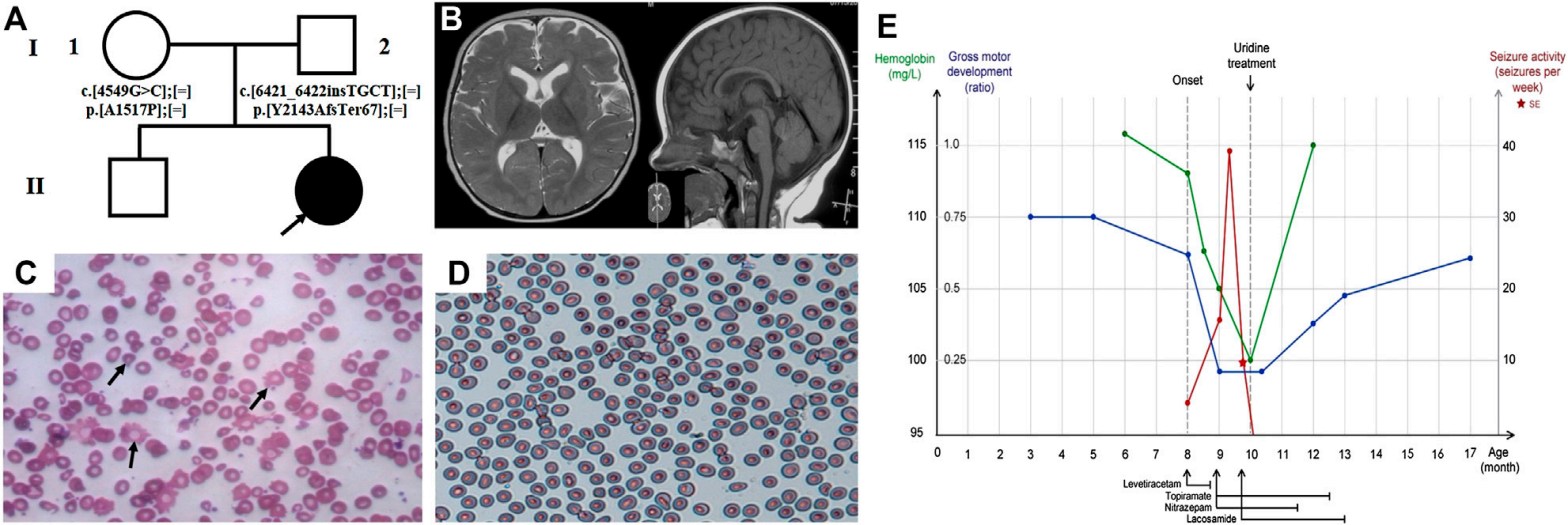
Clinical features and uridine response of patient 2 with CAD deficiency. **(A)** Family pedigree of patient 2. **(B)** Brain MRI at 8.5-months. Peripheral blood smear **(C)** before treatment and **(D)** after treatment. **(E)** Clinical overview during the course of disease. Gross motor development represented as the observed development age according to the Nelson Gross Motor Development Milestones divided by the patient’s biological age.

Patient 2 is a girl currently 17-months of age, born at term to a healthy nonconsanguineous family and fed by both breast and formula milk (Figure 2A). She presented with recurrent epileptic seizures at the age of 8-months. The seizures mainly presented focally with secondary generalisation. She experienced one episode of status epilepticus. When her seizures attack, her head and eyes tilted to the right, with salivating and lips turning blue, sometimes accompanied by ipsilateral or bilateral stiffness or waving of limbs, and usually lasting for one to 10 min. A mild developmental delay was noted prior to seizure onset, as she was able to raise her head at 3-months, roll over at 5-months, sit alone at 8-months, and she lacked visual fixation and following responses at 8-months. After seizure onset, she developmentally regressed and was unable to roll over or sit unsupported. Furthermore she developed hypotonia. The brain MRI performed at 8.5-months was unremarkable (Figure 2B). The ictal VEEG at 9-months recorded sharp (spike) slow wave discharges while monitoring a seizure attack (eyes turned right, salivating, with right limbs stiffness and shaking), and the interictal EEG documented sharp and spike slow wave in the bilateral occipitotemporal lobe (Supplementary Figure S1C). Her whole blood cell count indicated mild anaemia (haemoglobin 10–10.5, reference 11–16 g/dl; mean corpuscular volume 79.4–82.9, reference range 80–100 fl) and the peripheral blood smear showed abnormal erythrocytes of varying size and morphology, suggestive of anaemia with anisopoikilocytosis (Figure 2C). VEP performed at 8-months suggested an optic nerve conduction block, with severe prolongation of binocular P2 latency. She responded poorly to multiple AEDs, including LEV (10 mg/kg day, withdrew due to suspicious allergy), TPM (5.5 mg/kg day), LCM (5.5 mg/kg day), and NZP (0.15 mg/kg day). She achieved a short seizure free period for approximately 2-weeks at the first onset after receiving an intramuscular injection of phenobarbital, however seizures recurred up to almost 20 times a day thereafter. 2-months after the first seizure, and 6-days after identification of the CAD variants, supplementation with oral uridine (100 mg/kg daily) commenced. This led to the prompt cession of seizures by treatment day two. She stopped taking AEDs (LEV, NZP, TPM and LCM in order) approximately 4.5-months after commencing uridine, and has remained seizure-free during the 7.5-months follow-up period to-date. Moreover, she demonstrated dramatic improvement in her psychomotor development, as she can sit alone and has become more responsive and able to fix and follow objects. A sequential interictal VEEG at 1-year of age recorded no epileptiform discharge, except for few atypical sharp waves in the left posteromedial temporal lobe during sleep without epileptiform discharge (Supplementary Figure S1D). Routine blood tests and the peripheral blood smear both normalised (Figure 2D). A re-examined VEP at 13-months of age demonstrated normalized amplitude of P100 and latency. Moreover, she has made further significant progress in global development, and is now able to walk with assistance and speak one to two words, such as “mama” and “I want.” Her clinical course is depicted in Figure 2E.

No adverse side effects of uridine administration were observed in either case. In-keeping with previous reports, despite CAD deficiency secondarily impairing glycosylation, urine purines and pyrimidines (including uracil, uric acid, hypoxanthine, and xanthine) and urine orotic acid testing prior to uridine supplementation were unremarkable, and urine purine testing showed only mild elevation of guanosine, deeming these biomarkers unsuitable in as biomarkers for the detection of CAD deficiency.

**FIGURE 3:**
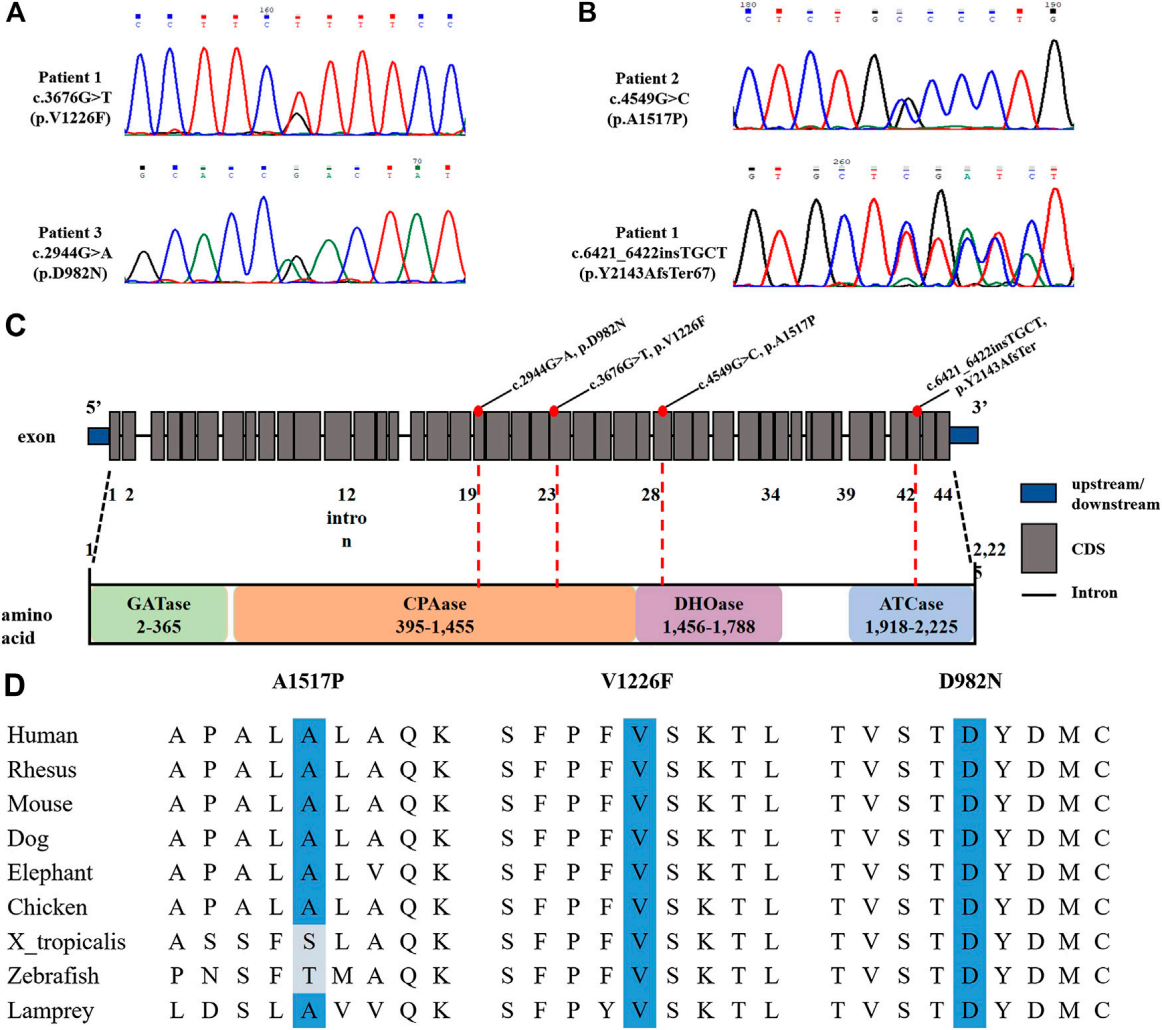
Gene results of two patients with CAD deficiency. **(A**,**B)** Sanger sequencing of our two cases demonstrated CAD compound heterozygous variants. **(C)** Location of CAD variants. **(D)** Evolutionary conservations of the missense mutations (A1517, V1226, and D982).

Genetically, compound heterozygous variants were identified in both patients in CAD (NM_004341) by trios-WES. Sanger sequencing was performed to confirm segregation by biparental transmission. In patient 1, the identified variants, c.3676G>T (p.V1226F) and c.2944G>A (p.D982N), were both interpreted as VUS (variant of unknown significance). As for patient 2, the identified variant, c.4549G>C (p.A1517P), was interpreted as likely pathogenic, while c.6421_6422insTGCT (p.Y2143AfsTer67) was predicted as VUS (Figures 3A,B). The CADD score, an in silico predictor of variant pathogenicity, was high in all missense variants (26.1, 30.0, and 24.4 for the p.V1226F, p.D982N, and A1517P, variants, respectively). The gnomAD allele frequency of the above variants were not listed, indicating the variants to be exceptionally rare. The pathogenicity of the variants was interpreted in accordance with the guidelines of the American College of Medical Genetics and Genomics (ACMG) (Richards et al., 2015). The human CAD gene consists of 44 exons and encodes four functional domains (GATase, CPAase, DHOase, and ATCase) that catalyse the first steps of *de novo* pyrimidine biosynthesis. The location of the novel variants in functional domains and their high-degree of conservation is depicted in (Figure 3C). According to sequence alignment, the amino acids A1517, V1226, and D982 are highly conserved across species, indicating evolutionary importance (Figure 3D). The mutation sites of the collectively described *CAD* mutations to date are distributed across all four functional domains of CAD, and include nonsense, splicing-site, and missense mutations, with no indication of specific phenotype-genotype correlation dependent on genomic position. They are all predicted to result in altered tertiary protein configuration and deteriorated protein function.

## Discussion

Here we recapitulate the clear clinical picture of CAD deficiency previously reported, with the co-occurrence of drug-resistant epilepsy, mostly focal or with generalization, developmental delay with regression, and anaemia with anisopoikilocytosis. Treatment with uridine supplementation was commenced despite the reported variants classification as VUS according to the ACMG criteria. Each of these phenotypes was responsive to supplementation, highlighting the need to act immediately upon discovery of such variants in treatable disease genes, where there is no known risk of side effects. Importantly, this targeted treatment allowed us to stop conventional AEDs with known and significant side effect profiles.

Furthermore, due to recognition and diagnosis early in the natural course of the disease, we do not report the typical neuroradiological sequelae of progressive cerebral and cerebellar atrophy described in the advanced stage of disease (Koch et al., 2017). While of four previously described cases where uridine treatment was not implemented, two died in early childhood after succumbing to their neurodegeneration and in the two surviving patients, uridine treatment was applied with a delay of 15-months and 58-months from onset to treatment, respectively (Koch et al., 2017). Monitoring for future evolution of the brain MRI in our cases will require long-term follow-up to determine whether progressive cerebral and cerebellar atrophy will develop as expected in the late stage of disease. However, given their remarkable clinical improvement to-date, we believe prompt uridine supplementation may have prevented these complications.

Moreover, the clinical investigations suggest an additional phenotype in CAD deficiency, as visual impairment due to optic nerve conduction block was demonstrated by VEP in both patients. As with the typical triad of clinical phenotypes, this phenotype is ameliorated by uridine supplementation, as patients demonstrate improvement in pursuit of objects and in sequential VEP assessment following treatment. A patient recently reported by Ling Zhou et al. presented with reduced visual acuity and was diagnosed with strabismus at 2 years old, also here both were improved by uridine supplementation within approximately 1 year (Zhou et al., 2020).

As the natural course has shown to be lethal in early childhood without treatment, these cases highlight the dramatical efficiency and paramount importance of early CAD deficiency recognition and the need to act immediately, even without a (definite) genetic diagnosis. We do, however, appreciate the long-term evolution is not known, and will require a longer period of follow-up. To conclude, these case reports reinforce CAD deficiency as a treatable neurometabolic disorder and emphase the need for a biomarker or genetic new born screening for early identification.

## Data Availability Statement

The raw data supporting the conclusions of this article will be made available by the authors, without undue reservation.

## Ethics Statement

Written informed consent was obtained from the minor(s)’ legal guardian/next of kin for the publication of any potentially identifiable images or data included in this article.

## Author Contributions

LZ, JD, and SS analysed the data and drafted the manuscript. JZ and HL collected and analysed the data. FF and HP critically revised and gave final approval for publication of the paper. Written consent for publication was obtained from the patients.

## Funding

The work was supported by special project for capital health development (No. 2018-2-2096), by Prevention and Control of Major Chronic Non-Communicable Disease (No. 2016YFC1306203) and by the BMBF through the European network of mitochondrial disorders (GENOMIT, No. 10GM1603), by Cultivation Fund Project of the National Natural Science Foundation in Beijing Children’s Hospital, Capital Medical University (GPQN201915), and Children’s Medicine Research Project of Beijing Children’s Hospital, Capital Medical University (YZQN202010).

## Conflict of Interest

The authors declare that the research was conducted in the absence of any commercial or financial relationships that could be construed as a potential conflict of interest.
